# Formation and Toxicity of Soluble Polyglutamine Oligomers in Living Cells

**DOI:** 10.1371/journal.pone.0015245

**Published:** 2010-12-28

**Authors:** Patrick Lajoie, Erik Lee Snapp

**Affiliations:** Department of Anatomy and Structural Biology, Albert Einstein College of Medicine, Bronx, New York, United States of America; University of Milano-Bicocca, Italy

## Abstract

**Background:**

Aggregation and cytotoxicity of mutant proteins containing an expanded number of polyglutamine (polyQ) repeats is a hallmark of several diseases, including Huntington's disease (HD). Within cells, mutant Huntingtin (mHtt) and other polyglutamine expansion mutant proteins exist as monomers, soluble oligomers, and insoluble inclusion bodies (IBs). Determining which of these forms constitute a toxic species has proven difficult. Recent studies support a role for IBs as a cellular coping mechanism to sequester levels of potentially toxic soluble monomeric and oligomeric species of mHtt.

**Methodology/Principal Findings:**

When fused to a fluorescent reporter (GFP) and expressed in cells, the soluble monomeric and oligomeric polyglutamine species are visually indistinguishable. Here, we describe two complementary biophysical fluorescence microscopy techniques to directly detect soluble polyglutamine oligomers (using Htt exon 1 or Htt^ex1^) and monitor their fates in live cells. Photobleaching analyses revealed a significant reduction in the mobilities of mHtt^ex1^ variants consistent with their incorporation into soluble microcomplexes. Similarly, when fused to split-GFP constructs, both wildtype and mHtt^ex1^ formed oligomers, as evidenced by the formation of a fluorescent reporter. Only the mHtt^ex1^ split-GFP oligomers assembled into IBs. Both FRAP and split-GFP approaches confirmed the ability of mHtt^ex1^ to bind and incorporate wildtype Htt into soluble oligomers. We exploited the irreversible binding of split-GFP fragments to forcibly increase levels of soluble oligomeric mHtt^ex1^. A corresponding increase in the rate of IBs formation and the number formed was observed. Importantly, higher levels of soluble mHtt^ex1^ oligomers significantly correlated with increased mutant cytotoxicity, independent of the presence of IBs.

**Conclusions/Significance:**

Our study describes powerful and sensitive tools for investigating soluble oligomeric forms of expanded polyglutamine proteins, and their impact on cell viability. Moreover, these methods should be applicable for the detection of soluble oligomers of a wide variety of aggregation prone proteins.

## Introduction

Numerous cellular proteins are regulated by interconversion between monomeric and oligomeric states. Distinguishing the different forms in live cells is challenging and requires the use of biophysical fluorescence techniques, including Förster Resonance Energy Transfer (FRET) and Fluorescence Correlation Spectroscopy (FCS) [1], [2]or enzymatic reporters that amplify a signal from weak protein-protein interactions, such as yeast two hybrid or split luciferase systems [3], [4]. While these methods can detect relative levels of oligomers, the methods do not distinguish the functional importance of monomeric and oligomeric species, which often co-exist in cells. For example, if a cytotoxic protein can form soluble oligomers, is the oligomeric species necessarily cytotoxic? The problem is especially relevant in Huntington's disease (HD), other polyglutamine expansion diseases, and aggregation-prone protein diseases in general. Knowing whether monomers, soluble oligomers or both forms are cytotoxic will impact therapeutic strategies- i.e. which form of the protein to target.

HD is an autosomal dominant neurodegenerative disease correlated with the expression of a mutant form of the huntingtin protein (Htt). This ubiquitously expressed large protein contains 3144 amino acids and an uninterrupted series of CAG repeats that are translated into a polyglutamine (polyQ) tract. Fewer than 36 CAG repeats produces no phenotype. In contrast, individuals with a copy of mutant Htt (mHtt) containing a pathogenic length of 36 to 150 CAG repeats have HD [5]. The age of pathology onset correlates inversely with the number of repeats [6]. HD is one of nine described polyQ diseases. Expression of exon 1 of mHtt is sufficient to promote development of severe disease symptoms in mice, resembling the ones observed in HD [7]. Exon 1 includes the first 67 amino acids of full length Htt with an internal stretch of a variable number of glutamines. Many HD studies suggest a key feature distinguishing wildtype (wt) and mHtt N-terminal fragments (including exon 1) is the tendency of the mutant protein to irreversibly aggregate into SDS-insoluble cytoplasmic amyloid-like fibrils termed inclusion bodies (IBs) [8], [9]. The role of the IBs remains controversial. While IBs have been associated with neuronal cell death [10], [11], [12], [13], other studies find cells die without ever forming IBs and correlate IBs with increased cell survival [14], [15], [16], [17], [18], [19]. IBs may act as a cellular coping mechanism to sequester and detoxify mHtt.

The increased tendency of mHtt to aggregate may represent an exaggerated behavior of an inherent property of wt Htt. Some biochemical data suggest wt Htt undergoes physiologic oligomerization in a regulatable manner [20]. For example, homo-oligomerization of wt Htt fragments can be increased by overexpression of p21 activated kinase (Pak1) [21]. Other live cell studies did not detect significant oligomerization of wt Htt^ex1^, even with sensitive biophysical techniques such as FRET [18]. The functional consequences of oligomerization of wt Htt remain unclear.

Oligomerization of mutant polyQ proteins appears to be detrimental to cells [18]. Several studies have employed inhibitory molecules and antibodies to block polyQ oligomer formation and observed a corresponding decrease in cell death [22], [23], [24], [25], [26], [27], [28], [29], [30], [31], [32]. Thus, the soluble oligomers forming prior to IBs may represent the toxic species in HD [33]. Yet, other studies have raised the potential for the toxicity of conformational variants of the monomeric species of polyQ proteins [26], [34]. The conformational hypothesis suggests reagents that block oligomerization may also be sterically obscuring toxic protein domains on monomers [33].

To better define the relevant species for polyglutamine toxicity, it would be useful to directly monitor both monomeric and oligomeric species, preferably in intact individual cells that could also be assayed for susceptibility to death. In addition, a method for increasing the cellular levels of soluble oligomers could more directly address the relative toxicities of Htt^ex1^ species. It was demonstrated that various mutant polyQ proteins (including mHtt^ex1^) can form soluble oligomers before IBs appear [18]. FRET approaches have successfully quantified interactions of mHtt^ex1^ in IBs in cells [18], [35]. However, detection of FRET signals for the soluble mHtt^ex1^ fraction has yielded conflicting results [18], [35]. FCS performed on lysates of cells expressing expanded polyQ-GFP also detected formation of soluble oligomers [30]. Detecting and characterizing the contributions of mutant polyglutamine monomers and oligomers in cells is fundamental to the understanding of polyglutamine diseases. Therefore, we sought to develop new approaches to monitor polyglutamine oligomer formation in living cells.

## Results and Discussion

### Experimental design

Two different approaches were employed to quantitate global changes in the monomeric mHtt pool and detect soluble oligomers in live cells. To study changes in the total population of mHtt^ex1^, we measured changes in protein mobility, and by extension molecular size, with Fluorescence Recovery after photobleaching (FRAP). For FRAP, GFP-tagged proteins are expressed in live cells and imaged with a scanning confocal microscope. A discrete region of interest (ROI) in a cell is irreversibly photobleached with high intensity laser light. Movement of unbleached fluorescent molecules into the ROI is quantitated over time and analyzed to determine the diffusion coefficient (*D*) of the fluorescent molecule [36]. *D* (µm^2^/s) is inversely proportional to environmental viscosity and the size of the molecule (hydrodynamic radius or R_h_) or an associated molecular complex [37]. Previously, FRAP analysis revealed that polyQ proteins, when incorporated into IBs, exhibit exceptionally low mobility [38]. However, these results provide no indication of the mobility of the polyQ protein prior its incorporation into IBs. While FRAP can report changes in molecular size, it provides no information on the composition of molecular complexes. Decreased mobility of mHtt proteins could represent mHtt^ex1^ oligomers, assemblies with other cellular proteins, such as chaperones or a mixture of both [39].

To specifically detect Htt oligomers, we employed split-GFP technology, also termed bimolecular fluorescence complementation (BiFC). Two non-fluorescent fragments of green fluorescent protein (GFP) can associate to form a single fluorescent GFP, but only when they are fused to two proteins capable of interacting with each other [40], [41], [42], [43]. Split-GFP is sufficiently sensitive to report weak or transient interactions even for small populations of interacting proteins that could be otherwise obscured in sensitized emission FRET experiments [41]. The irreversible nature of the split-GFP interaction also allows trapping of otherwise transient interactions [44]. Therefore, this feature can be used to enrich the pool of oligomeric Htt^ex1^in living cells [45]. Using FRAP and split-GFP, we sought to detect changes in soluble Htt^ex1^ oligomer levels in live cells and correlate oligomer formation with mHtt^ex1^ toxicity.

### mHtt polyQ expansion and formation of IBs in neuronal cells

**Figure 1:**
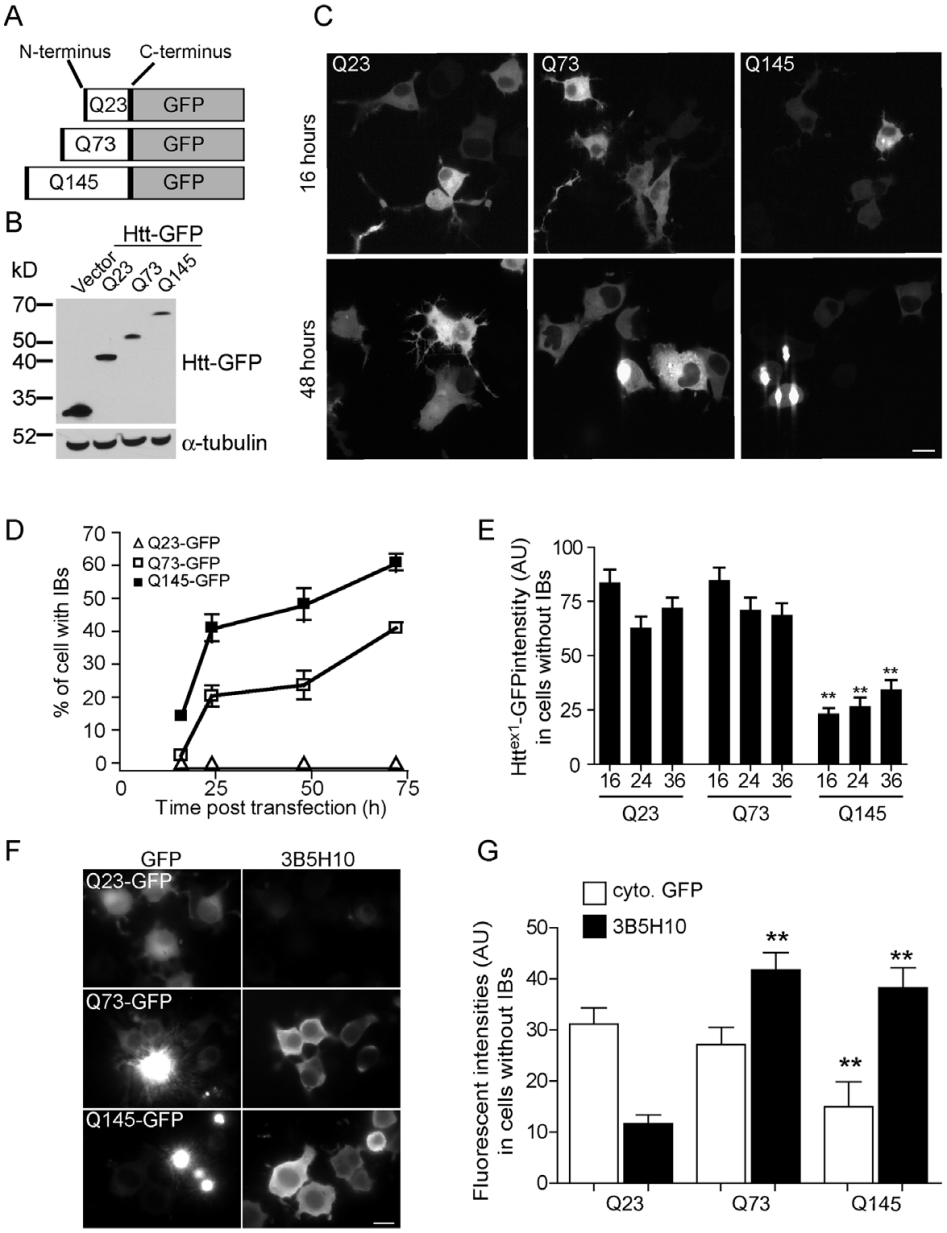
Increased number of polyQ repeats correlates with formation of Htt^ex1^-GFP inclusion bodies. (**A**) Diagram showing the Htt^ex1^-GFP constructs containing 23, 73 and 145 polyQ repeats. (**B**) Western blot showing the relative sizes of the Q23, 73 and 145 Htt^ex1^-GFP constructs relative to the empty GFP vector. (**C**) Representative fluorescent images of N2a cells transfected with Q23, 73 or 145 Htt^ex1^-GFP for 16 and 48h. Images were collected with the same settings. (**D**) Quantitation of the percentage of cells containing IBs for various time points following transfection with Q23, 73 or 145 Htt^ex1^-GFP. (**E**) Quantitation of the Q23, 73 and 145 Htt^ex1^-GFP mean fluorescent intensities at indicated times in cells without IBs. Each histogram bar is a mean of values for 30 or more cells. (**F**) Fluorescent images of N2a cells expressing Htt^ex1^-GFP constructs containing 23, 73 or 145 polyQ repeats and immunofluorescently labeled with 3B5H10 antibody. (**G**) Cytoplasmic GFP intensity for individual cells is quantitated and plotted against the 3B5H10 intensity only in cells not presenting IBs. Images were collected with the same settings Bar = 20 µm.

To study mHtt^ex1^ aggregation in living cells, Htt^ex1^ sequences containing 23, 73 or 145 CAG repeats were fused to monomeric GFP (Figs. 1A and B). When transfected into differentiated N2a cells, Q73 and 145 mHtt^ex1^-GFP exhibited diffuse cytoplasmic fluorescence and gradually developed visible IBs. In contrast, the wt form, Q23 Htt^ex1^-GFP, only exhibited a diffuse cytoplasmic distribution, similar to previous reports (Fig. 1C and D) [46]. The Q145 construct developed IBs more quickly and in substantially more cells than the Q73 construct (Fig. 1D). Interestingly, the fluorescence intensity of the soluble Q145-GFP measured in cells not presenting IBs was significantly lower than the Q73 mutant and the wt Q23 Htt^ex1^-GFP at multiple times post-transfection (Fig. 1E). This difference may reflect an increased propensity of the Q145 protein to be degraded by the proteasome or other degradation pathways, such as autophagy [47], [48], [49]. The fluorescence result is consistent with the observed comparatively low levels of Q145-GFP in the whole cell lysate immunoblot (Fig. 1B).

Potentially pathogenic soluble cytoplasmic forms of Q73 and Q145 mHtt^ex1^-GFP constructs can also be detected using the 3B5H10 antibody, which exclusively stains *soluble* cytoplasmic mHtt, but not IBs (Fig. 1F) [34], [50]. The 3B5H10 antibody staining intensity was significantly higher for mutant Q73 and Q145 constructs relative to the Q23 construct (Fig. 1G) Recently, this antibody was shown to specifically recognize a potentially toxic conformation of mHtt monomers [33], [34]. Interestingly, even when cells expressing Q145 Htt^ex1^-GFP presented relatively low cytoplasmic GFP intensity, these same cells exhibited robust 3B5H10 staining, comparable with Q73 expressing cells (Fig. 1G). The poor correlation between GFP intensity and 3B5H10 staining likely reflects the affinity of the antibody for extended polyglutamine stretches or for a specific conformation of soluble mHtt constructs.

### FRAP analysis reveals the incorporation of mHtt^ex1^ into soluble complexes

**Figure 2:**
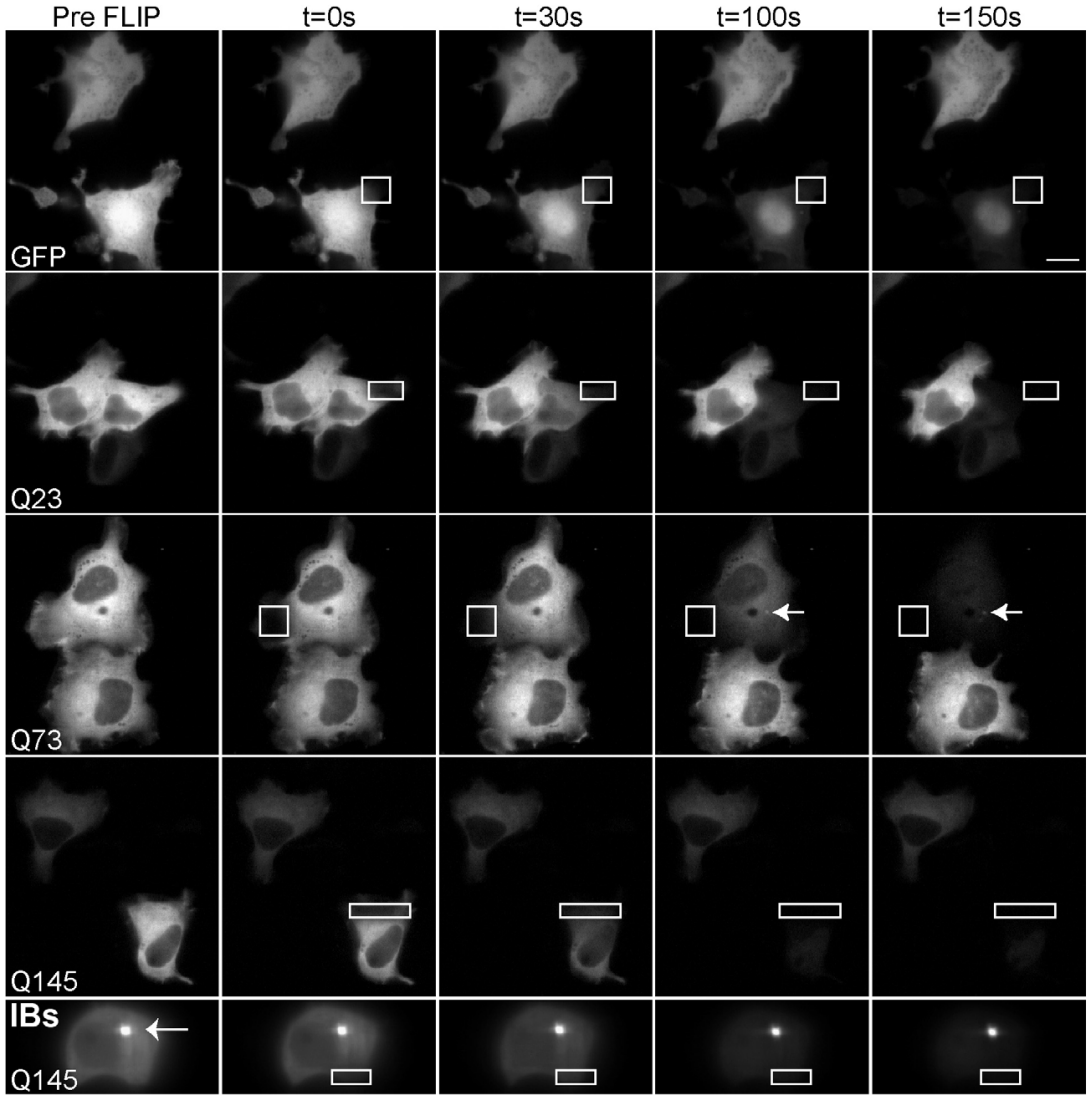
Mobility of Htt^ex1^-GFP constructs. FLIP analysis of Htt^ex1^-GFP mobility in the cytoplasm of N2a cells transfected for 24 h. Repetitive photobleaching of cells within the cytoplasm, in a small ROI (white outline box), was performed. The 0s image represents the first image after the first photobleach. The same ROI was photobleached every 5 seconds. By 150 s, nearly all fluorescence had been depleted from all of the cytoplasm, whereas adjacent cell fluorescence was unaffected. FLIP reveals the presence of small stable IBs (73, arrowhead). When IBs were present, repetitive photobleaching depleted the cytoplasm fluorescence without significantly affecting the IBs fluorescent intensity (bottom row; arrowhead). Bar = 20 µm.

To determine whether the various constructs were suitable for FRAP analysis, we first established whether the GFP-tagged mHtt^ex1^ constructs were mobile in the cytoplasm and, thus, capable of diffusing. In Fluorescence Loss in Photobleaching (FLIP) an ROI is repeatedly photobleached and alternately imaged. All fluorescence within the cell will be depleted if the fluorescent molecules outside of the ROI are mobile and can pass through the ROI during the time course of the experiment [36]. FLIP experiments revealed that only the soluble pool of mHtt is mobile while the IBs appear to be very stable structures (Fig. 2), as previously described for mHtt and other polyQ proteins [51], [52], [53]. Similar mobility results were observed in photoactivation experiments, in which mHtt^ex1^ was fused to photoactivatable GFP and optically highlighted (Fig. S1).

**Figure 3:**
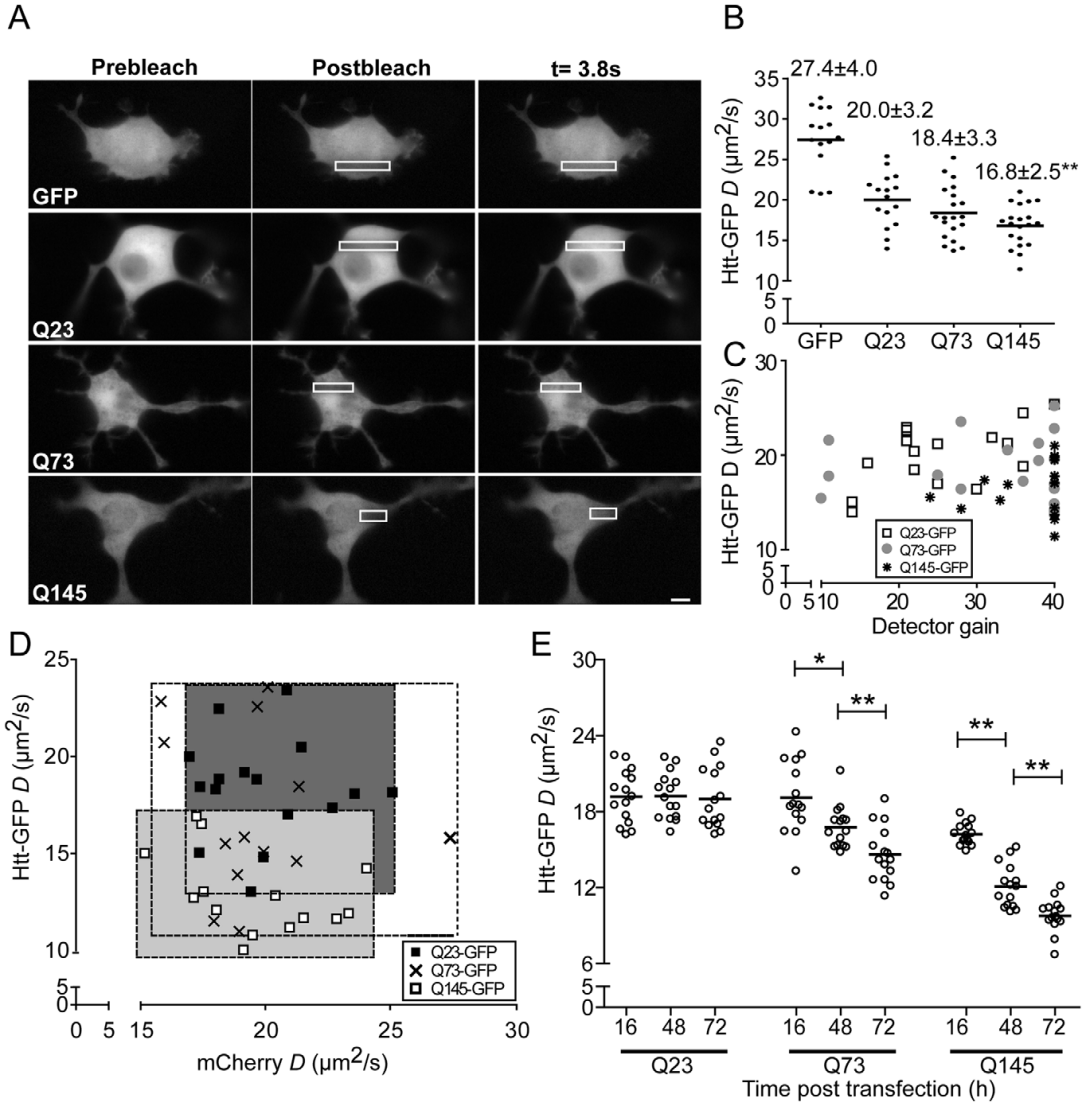
FRAP analysis reveals incorporation of Htt^ex1^-GFP mutants into microcomplexes. (**A**) Fluorescent images of N2a cells transiently transfected 16 h (to minimize the number of IBs) with Htt^ex1^-GFP constructs containing 23, 73 or 145 polyQ repeats arte shown before (prebleach), immediately after (t = 0) and after recover (t = 3.8 s). (**B**) *D* values (µm^2^/s) of single N2a cells transiently transfected with Htt^ex1^-GFP constructs containing 23, 73 or 145 polyQ repeats and analyzed by FRAP. (**C**) Plot of *D* values (µm^2^/s) and camera gain settings for N2a cells transiently expressing Q23, Q73 or Q145 Htt^ex1^-GFP. (**D**) *D* values (µm^2^/s) of single N2a cells transiently cotransfected with Htt^ex1^-GFP constructs containing 23, 73 or 145 polyQ repeats and cytosolic mCherry and analyzed by FRAP. (**E**) *D* values (µm^2^/s) for N2a cells transiently expressing Q23, 73 or 145 Htt^ex1^-GFP for 16, 48 and 72 h. Bar = 20 µm.

Next, we used FRAP to determine whether we could detect quantitative differences in the mobilities of soluble polyglutamine constructs with increasing numbers of polyQ repeats. No detectable fluorescence recovery was observed when IBs were photobleached during the brief time course of the experiment (data not shown). We predicted all of the soluble Htt^ex1^-GFP constructs would be sufficiently large to diffuse more slowly than cytoplasmic GFP alone. It was unclear whether the constructs would diffuse significantly differently from each other. Purified polyQ rapidly oligomerizes into 180 nm+ diameter aggregates in solution [54]. Such large structures would likely exhibit low, if any, mobility in cells [55]. Interestingly, full-length wt Htt with 15 polyQ repeats has a reported large diameter of 19 nm [56]. Soluble mHtt^ex1^ oligomers from cells are 4–50 nm in diameter [57]. Molecules larger than 20–30 nm are not expected to diffuse significantly within the cytoplasm [58]. In fact, some proteins as small as 14.4 nm appear effectively immobile in the cytoplasm [59]. Consistent with FLIP and photoactivation experiments, all of our Htt^ex1^-GFP constructs were mobile in the cytoplasm (Fig. 3A). However, the Q145 mHtt^ex1^-GFP exhibited significantly lower *D* values than the Q23 and Q73 Htt^ex1^-GFP proteins (Fig. 3B). Similar results were observed in two additional cell types that were transiently transfected and analyzed by FRAP (Fig. S2). No significant correlation between *D* (µm^2^/s) values and the expression level of Htt^ex1^-GFP (as measured by microscope detector gain) was observed (Fig. 3C).

While the sizes of the constructs could account for the mobility differences, diffusion is also inversely proportional to the environmental viscosity. Overexpression of Htt-GFP could potentially alter the cytoplasmic viscosity and confound interpretation of the changes in mHtt^ex1^-GFP *D* (µm^2^/s) values. To control for viscosity changes, we co-transfected N2a cells with the various Htt^ex1^-GFP vectors and an inert cytoplasmic reporter, mCherry. As in Figure 3B, we detected a reduction in the Q145 mHtt^ex1^-GFP mobility relative to the other constructs. However, no significant change occurred for the mCherry when co-expressed with any of the constructs (Fig. 3D). Thus, the observed change in mobility for Q145 mHtt^ex1^-GFP was not due to changes in cytoplasm viscosity. Therefore, we could calculate apparent size differences for the different constructs, using the Stokes-Einstein equation [37]. Assuming GFP has an R_h_ of 2.3 nm [60], the GFP fusions to Q23, Q75, and Q145 had R_h_ values of ∼3.15, 3.4, and 3.7 nm, respectively. When factoring in the size of GFP, Q145 is 1.4 fold larger than the Q23 construct based on primary sequence. With this in mind, the 1.2 fold difference in R_h_ suggests the constructs are monomeric or not assembled into larger complexes at early times post-transfection.

What happens to the sizes of the soluble constructs as cells begin to accumulate IBs? Previously, FCS analysis of cell lysates containing pure polyQ-GFP proteins revealed polyQ protein oligomers form and increase in size up to 8.5 fold over 72 h [30]. Similarly we observed the majority of Q73 and Q145 mHtt^ex1^-GFP protein mobilities decreased in mobility in cells while the Q23 Htt^ex1^-GFP *D* (µm^2^/s) values remain unchanged over 72 h (Fig. 3E). Thus, pathogenic polyglutamine expansion length constructs incorporate into mobile soluble complexes, distinct from IBs.

### Detection of Htt^ex1^ oligomers using split-GFP in IBs free cells

**Figure 4:**
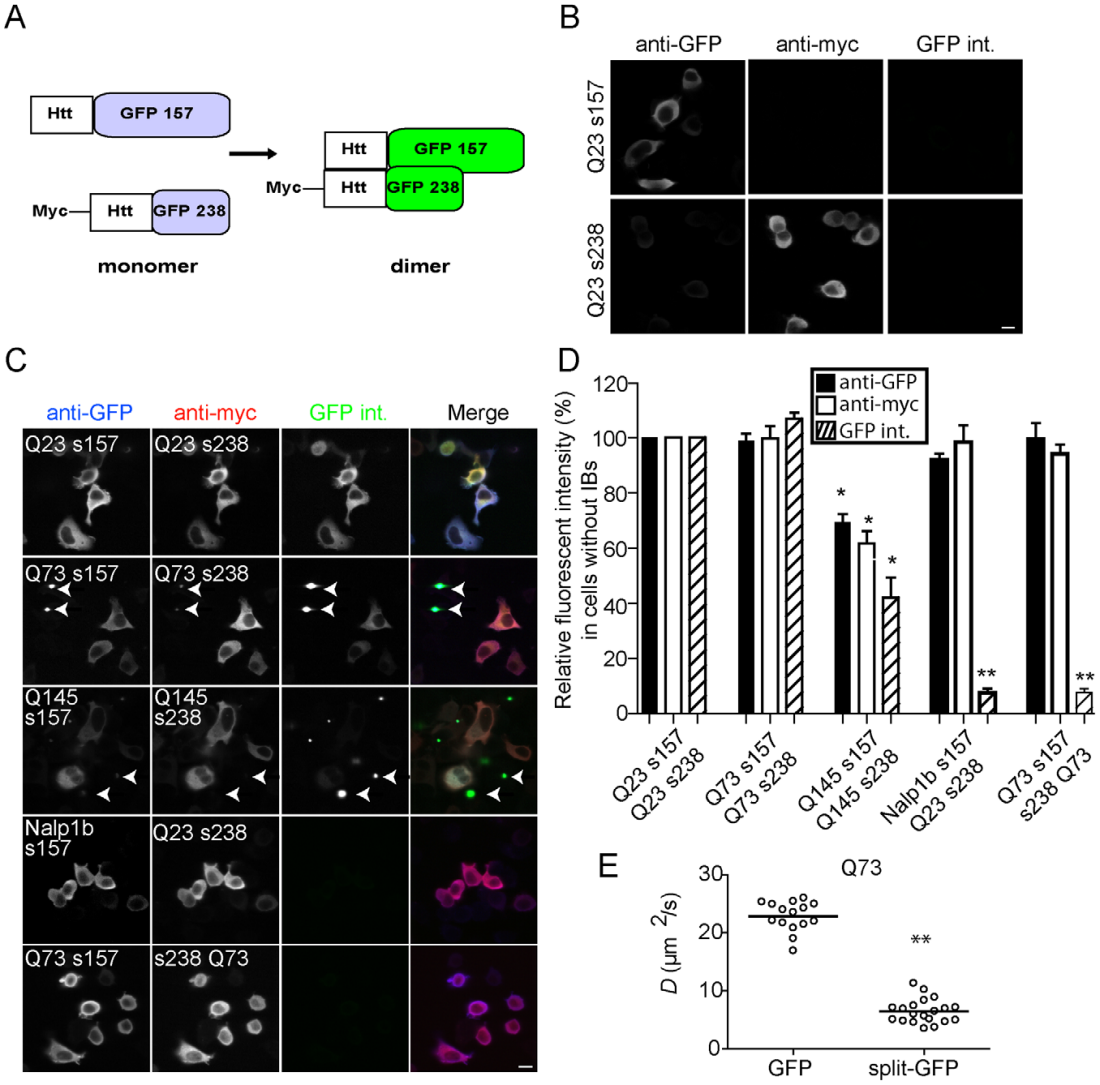
Visualization of Htt^ex1^-GFP oligomers using split-GFP. (**A**) Illustration of the fusion of wt and mHtt^ex1^ to either 157-GFP or 238-GFP. (**B**) Cells transfected with Q23 s157 or Q23 s238 separately, stained with anti-GFP or anti-myc, and imaged for GFP fluorescence intensity (GFP int.). (**C**) Representative images of N2a cells transiently transfected with Htt^ex1^Q23 157-GFP+Htt^ex1^Q23 238-GFP, Htt^ex1^Q73 157-GFP+mHtt^ex1^Q73 238-GFP, mHtt^ex1^Q145 157-GFP+mHtt^ex1^Q145 238-GFP, Nalp1b-157+Htt^ex1^Q23 238-GFP or mHtt^ex1^Q73 157-GFP+238-GFP mHtt^ex1^Q73. (**D**) Mean fluorescence intensities in cells without IBs are plotted and compared relative to Q23 means, which were set as 100%. (**E**) *D* values of single N2a cells transiently transfected with mHtt^ex1^Q73-GFP or -split GFP constructs and analyzed by FRAP. Bar = 20 µm. All fluorescence images were collected with the same settings.

Our FRAP results revealed incorporation of mHtt into increasingly larger complexes of unknown composition. To determine if at least some of these complexes contain mHtt oligomers, we turned to split-GFP technology to directly visualize soluble polyglutamine oligomer formation in living cells [61], [62], [63], [64], [65]. All three (Q23, Q73 and Q145 Htt^ex1^) constructs were fused at their COOH termini to split-GFP variants (1–157 and 158–238, we termed s157 and s238) [65] of superfolder GFP (Fig. 4A) [66]. A myc tag was also added to the NH_2_ terminus of Htt^ex1^ constructs inserted in the 158–258 SFGFP vector. When expressed separately in N2a cells, split-GFP vectors displayed no detectable GFP fluorescence (Fig. 4B). The proteins could be differentially labeled by immunofluorescence to monitor overall expression levels in the absence of GFP fluorescence (Fig. 4B). Htt s157-GFP is preferentially recognized by an anti-GFP antibody [67], while Htt s238-GFP is recognized by the anti-myc antibody (Fig. 4B). When expressing both pieces of split-GFP fused to the various Htt^ex1^ constructs, all three pairs exhibited GFP fluorescence (Fig. 4C). All three constructs displayed GFP signals higher than the background levels detected for transfection of Q23 Htt^ex1^ s238 co-transfected with the negative control cytoplasmic protein, Nalp1B tagged to s157 (Fig. 4C and D).

GFP fluorescence depended on the position of the split GFP. Co-transfection of cells with Q73 mHtt^ex1^ split-GFP constructs in which a COOH- terminal S157 Q73 construct was cotransfected with a Q73 construct with the s238 fused to the NH_2_ terminus resulted in no fluorescence (Fig. 4C). Our results are consistent with the FRET results of Takahashi et al., who reported only detecting FRET when the fluorescent proteins were placed on the same end of the two interacting mHtt^ex1^ proteins [18]. Thus, mHtt^ex1^ proteins must assemble in parallel to form oligomers.

As we also observed for the intact Q145 mHtt^ex1^-GFP fusion, the split-GFP constructs mean cytoplasmic GFP intensity measured in cells not presenting IBs was substantially lower than for Q73 (Fig. 4C and D). This may reflect increased targeting of Q145 for degradation. Remarkably, we detected a robust GFP intensity for the wt Q23 constructs, demonstrating the wt Htt^ex1^ can oligomerize [21]. We discuss the implications of this observation in the next section.

When compared to the expression level of the whole intact GFP construct, Q73 mHtt^ex1^ split-GFP fluorescence was significantly lower, even though the total level of soluble toxic mHtt is similar as measured with 3B5H10 levels by immunofluorescence (Fig. S3). These data indicate mHtt^ex1^ oligomers represent a minor population of the total mHtt^ex1^ pool. In theory, this is to be expected. Since 50% of Htt molecules fused to the same split-GFP moiety could form dimers and the resulting homodimers would be nonfluorescent, the split-GFP signal should only represent, ideally, 50% of the total pool of Htt oligomers. Furthermore, if the same total amount of split-GFP constructs relative to intact Q73 GFP is expressed, then the maximal possible split-GFP fluorescence signal is only 50% of intact GFP. Together, these two qualifications should make split-GFP cells only 25% as bright as intact GFP cells, at best. This assumes all of the split-GFP constructs interact to form fluorophores.

Next, we compared the mobilities of intact and split GFP Q73 constructs 16h after transfection, in cells without IBs. Q73 mHtt^ex1^-split-GFP exhibited a four fold decreased mobility relative to intact Q73-GFP (mean *D*± standard errors of 22.8±0.7 vs. 6.5±0.5) (Fig. 4E). This difference is significantly larger than would be anticipated for simply dimerizing the two proteins. Even if the molecules were arranged end to end, the predicted increase in R_h_ would only be two fold, with a corresponding two-fold decrease in *D*. Therefore, the split-GFP Q73 construct oligomer must contain multiple dimers or associate with cytoplasmic chaperones. Furthermore, as FRAP is an ensemble measurement, our results suggest a mean increase in oligomer complex size, which could easily include the 8.5 fold larger complexes reported in the FCS study [30].

### Incorporation of wt Htt^ex1^ into mHtt^ex1^ oligomers

Several lines of evidence suggest HD pathology is due to a gain of function of the mHtt as a result of the extended polyQ stretch [68]. However, an alternative hypothesis suggests the HD phenotype is due to loss of function of the wt Htt protein [69]. Consistent with this hypothesis, mHtt^ex1^ protein can recruit wt Htt^ex1^ into insoluble aggregates both *in vitro* and *in vivo* [70], [71], [72].

**Figure 5:**
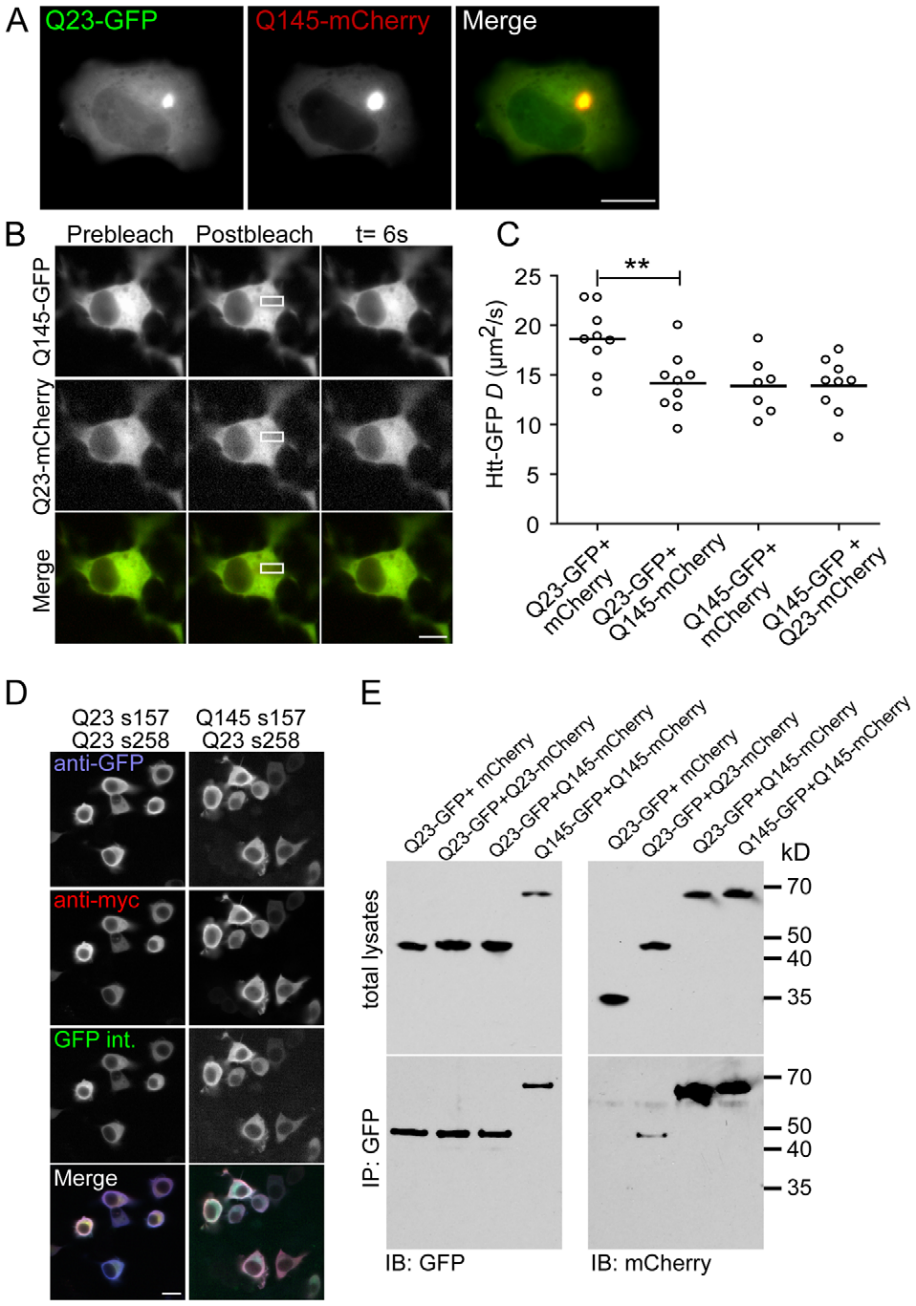
Incorporation of wt Htt^ex1^-GFP into mutant oligomers. (**A**) Representative fluorescent images of N2a cells cotransfected with Htt^ex1^Q23-GFP and Q145-mcherry for 48 h. (**B**) Fluorescent images of N2a cells transiently cotransfected with Htt^ex1^Q23-GFP and mHtt^ex1^Q145-mCherry constructs are shown before (prebleach), immediately after (t = 0) and after recover (t = 6s). (**C**) *D* values for N2a cells co-expressing Htt^ex1^Q23-GFP+mCherry, Htt^ex1^Q23-GFP+mHtt^ex1^Q145-GFP plus cytoplasmic mCherry, and mHtt^ex1^Q145-GFP+cytoplasmic mCherry and mHtt^ex1^Q145-GFP+Htt^ex1^Q23-mCherry. (**D**) Representative fluorescent images of N2a cells transiently transfected Htt^ex1^Q23 s157-GFP+Htt^ex1^Q23 s238-GFP, mHtt^ex1^Q145 s157-GFP+Htt^ex1^Q23 s238-GFP. All images were collected with the same settings for each channel. (**E**) N2a cells cotransfected with Htt^ex1^Q23-GFP+cytoplasmic mCherry, Htt^ex1^Q23-GFP+Htt^ex1^Q23-mCherry, Htt^ex1^Q23-GFP+mHtt^ex1^Q145-mCherry or mHtt^ex1^Q145-GFP+mHtt^ex1^Q145-mCherry. Lysates were incubated with anti-GFP-agarose beads and the lysates and coimmunoprecipitation are shown on immunoblots probed with anti-GFP and anti-mCherry. Bar = 20 µm.

It remains unclear whether wt binds directly to IBs or can interact with mHtt prior to incorporation into IBs. We investigated whether wt/mHtt^ex1^ soluble oligomers format in living cells. First, we confirmed our mHtt^ex1^ could sequester wtHtt^ex1^ into IBs. Q23 Htt^ex1^-GFP and Q145 mHtt^ex1^-mCherry were co-expressed in N2a cells. Wt Q23 was readily detectable with Q145 in the IBs (Fig. 5A). Next, we used FRAP to determine whether coexpression of wt and mHtt affected diffusion of both proteins (Fig. 5B). Importantly, we found that expression of Q145 mHtt^ex1^ significantly decreased Q23 Htt^ex1^-GFP mobility (Fig. 5C). Data from Figure 3D established oligomer formation does not generally slow the mobility of other proteins in the cytoplasm. Thus, the FRAP data in Fig. 5B suggest wt protein incorporates into larger soluble complexes in the presence of mHtt^ex1^. To test whether the complexes include oligomers of wt and mHtt^ex1^, we used the split-GFP approach to directly visualize hetero-oligomer formation between wt and mHtt. The robust fluorescent signal revealed the ability of mHtt to recruit wt protein into soluble oligomers (Fig. 5D). These results were subsequently validated with immunoprecipitations. When coexpressed in N2a cells, Q23 Htt^ex1^-GFP was able to pull down Q145 mHtt^ex1^-mCherry to a level similar to cotransfected Q145 Htt^ex1^-GFP with Q145 mHtt^ex1^-mCherry (Fig. 5E). Experiments were performed at 16 h posttransfection to minimize the number of IBs.

In contrast, little binding was detected between Q23 constructs fused to intact fluorescent proteins, Q23 Htt^ex1^-GFP and Q23 Htt^ex1^-mCherry. These data are consistent with a previous report of low FRET levels between wt Htt fragments [18], but contrast with our observation of Q23 Htt^ex1^ oligomerization when fused to split-GFP fusion proteins. The split-GFP interaction is irreversible [44], [73]. Therefore, we hypothesize our data reflect accumulation of otherwise reversible interactions between wt Htt^ex1^ monomers. Unlike interactions of wt Htt^ex1^ with mHtt^ex1^monomers, wt-wt Htt^ex1^ monomer binding events do not result in IBs formation [74]. Thus, split-GFP technology both detects and traps transient protein-protein interactions [44]. Our data reveal mHtt can and does sequester wt protein regardless of whether IBs form. Interestingly, an increased number of polyQ repeats in mHtt N-terminal fragments prevents wt fragments binding to Hip-1, leaving the latter free to form pro-apoptotic complexes [75]. Formation of wt and mHtt hetero-oligomers may contribute to this phenomenon. We speculate these previously difficult to detect interactions in cells are likely to be important to pathogenesis of polyglutamine diseases.

### Increased oligomerization correlates with increased cell death

In light of our findings, we asked if increased mHtt^ex1^ oligomerization affected IBs formation and mHtt^ex1^ toxicity. In their FRET study, Takashi and colleagues reported the soluble form of mHtt^ex1^ represents the toxic species in HD [18] and IBs are protective [14]. However, they did not observe a significant correlation between mHtt^ex1^ oligomer formation (FRET positive) and cell death when compared to FRET negative cells. The result suggests both mHtt oligomers and β-strand polyQ monomers are potentially toxic to cells. In addition, the conformation of mHtt^ex1^ may be a determinant of its toxicity [76].

**Figure 6:**
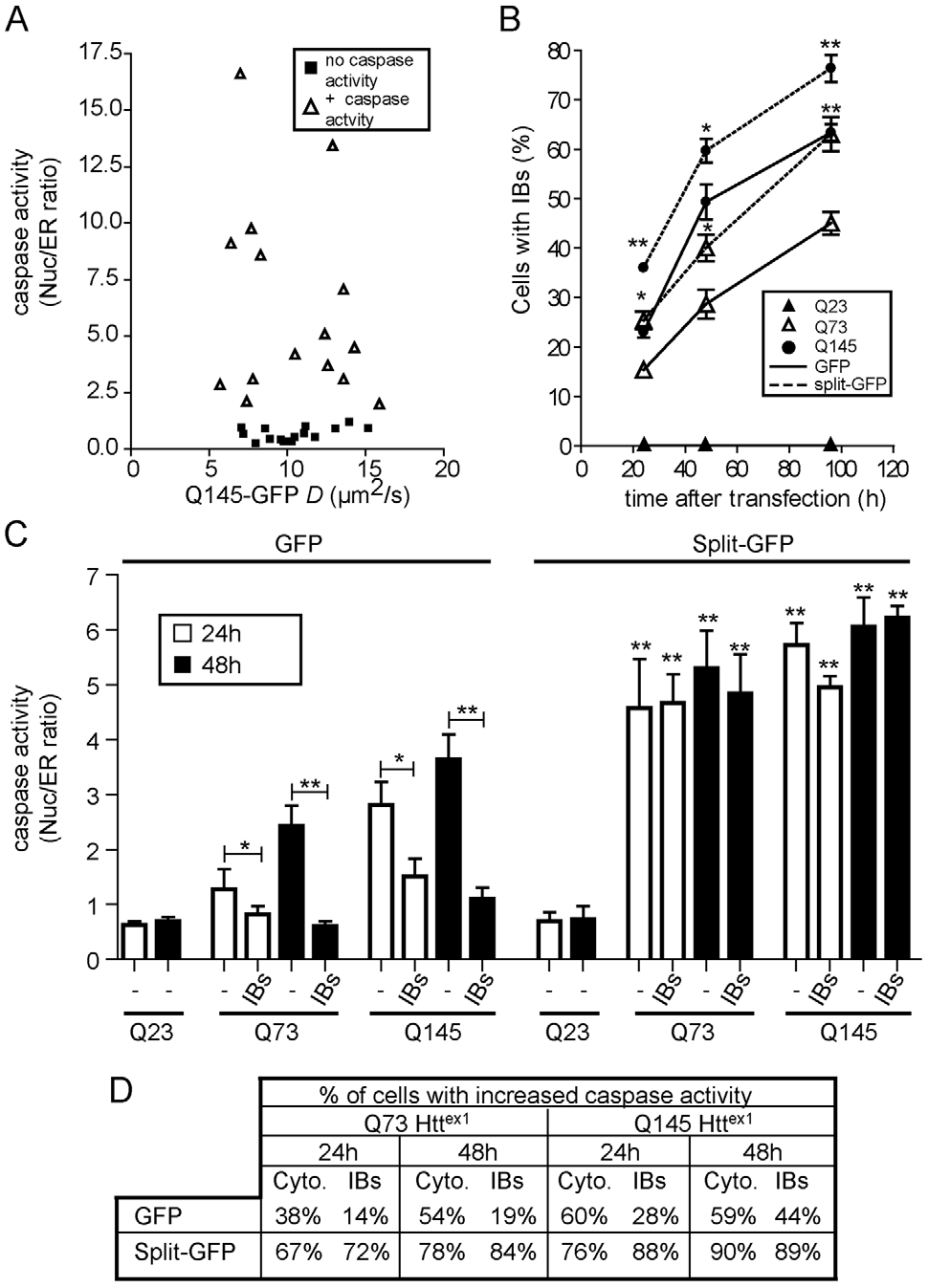
Fusion of mHtt to split-GFP results in increased IBs formation and cell death. (**A**) *D* values (µm^2^/s) for N2a cells transiently cotransfected with mHtt^ex1^Q145-GFP and ER-DEVD-tdTomato. (**B**) Quantitation of percentage of cells containing IBs for indicated times posttransfection with Htt^ex1^Q23, 73 or 145 fused to SFGFP or split-SFGFP constructs. n>215 cells. * p<0.05, ** p<0.005 compared to same length of polyQ fused to GFP. (**C**) N2a cells were cotransfected with Htt^ex1^-GFP or Htt^ex1^ split-GFP and ER-DEVD-tdTomato. Both aggregation and cell death were monitored at 24 and 48h posttransfection in cells without (-) or with IBs (inclusion bodies). Each histogram bar reports the mean caspase activity ± standard error. n>38 cells per condition. * p<0.05, ** p<0.005 compared to same parameter (+ or − IBs) for the same Htt^ex1^ construct fused to GFP unless specified. (**D**) Table of percentages of cells with caspase activity with or without IBs.

To test whether or not incorporation of mHtt into potentially oligomeric complexes impacts cell viability, we used our FRAP assay to determine if mHtt mobility correlates with neuronal cell survival. N2a cells were transfected with the live cell apoptosis reporter ER-DEVD [77]. The reporter localizes to the endoplasmic reticulum (ER). Upon induction of apoptosis with staurosporine, activated caspases cleave the DEVD peptide in the reporter, releasing it from the ER membrane, followed by translocation to the nucleus (Fig. S4). N2a cells were cotransfected with ER-DEVD-tdTomato and then Q145 mHtt^ex1^-GFP and analyzed by FRAP 48 h posttransfection. FRAP was performed on cells without (no caspase activity) and with nuclear localized reporter (+caspase activity) and Q145 mHtt^ex1^-GFP mobility was calculated. Consistent with the mHtt FRET reporter results, no relationship was observed between mHtt mobility and cell death (Fig. 6A) [18]. The FRAP results do not resolve the relative contributions of monomeric and oligomeric mHtt^ex1^, each of which may be equally toxic.

To investigate the cellular consequences of increased levels of mHtt^ex1^ oligomers, we exploited the irreversible nature of the split-GFP complementation system. We hypothesized the split GFP would increase the concentration of oligomers in two ways. First, the irreversibility of split-GFP makes it a powerful trap for normally transient interactions [44]. Second, the nonproductive dimers formed by constructs containing the same split-GFP fragment will not be trapped and, should, associate at some rate with a complementary split-GFP fragment to be trapped. Thus, split-GFP should increase the fraction of detectable (by GFP fluorescence) and irreversible dimers of mHtt^ex1^. Indeed, when run on a native polyacrylamide gel, Q23 Htt^ex1^ fused to split-GFP constructs exhibited formation of higher molecular weight complexes when compared to the Htt^ex1^ fused to intact GFP migrated much more rapidly as a small, presumably monomeric species (Fig. S5). Interestingly, Q23-s157 was unable to induce the formation of higher oligomers when expressed with Q23 fused to intact GFP (Fig S5). This result confirmed the ability of split-GFP to stabilize otherwise transient interactions. Based on these data, we then asked if trapping mHtt^ex1^-mHtt^ex1^ interactions would increase IBs formation. Fusion of mHtt^ex1^ to split-GFP enhanced both the rate of IBs formation and the number of cells with IBs, relative to mHtt fusions to intact GFP (Fig. 6B). Our data do not exclude the possibility that monomeric mHtt^ex1^ may also be recruited to IBs [33].

Next, we asked whether increased levels of soluble mHtt oligomers enhance cytotoxicity of polyglutamine proteins. Following transfection, levels of plasmid expressed proteins should steadily increase. Higher protein concentrations should increase the probability of protein oligomerization and aggregation. Cells expressing mHtt^ex1^ fused to intact GFP exhibited increasing levels of caspase activity with increasing time post-transfection (Fig. 6C). No caspase activity was observed for cells expressing Q23-GFP (Fig. 6C). When Htt-GFP expressing cells were categorized as having or lacking IBs, a significant trend was revealed. Consistent with previous reports, cells had reduced apoptotic activity when IBs were present compared to cells with diffuse cytoplasmic mHtt, [14], [18] (Fig. 6C). The percent of cells with caspase activity remained low over 48h for Q73-GFP expressing cells containing IBs, but increased about 50% for cells expressing Q145-GFP (Fig. 6D). These data suggest the presence of IBs could help protect cells from mHtt cytotoxicity and are consistent with previous reports [14], [18]. As with the FRAP data, it remained unclear whether soluble monomers or oligomers preferentially affected mHtt-GFP cytotoxicity.

**Figure 7:**
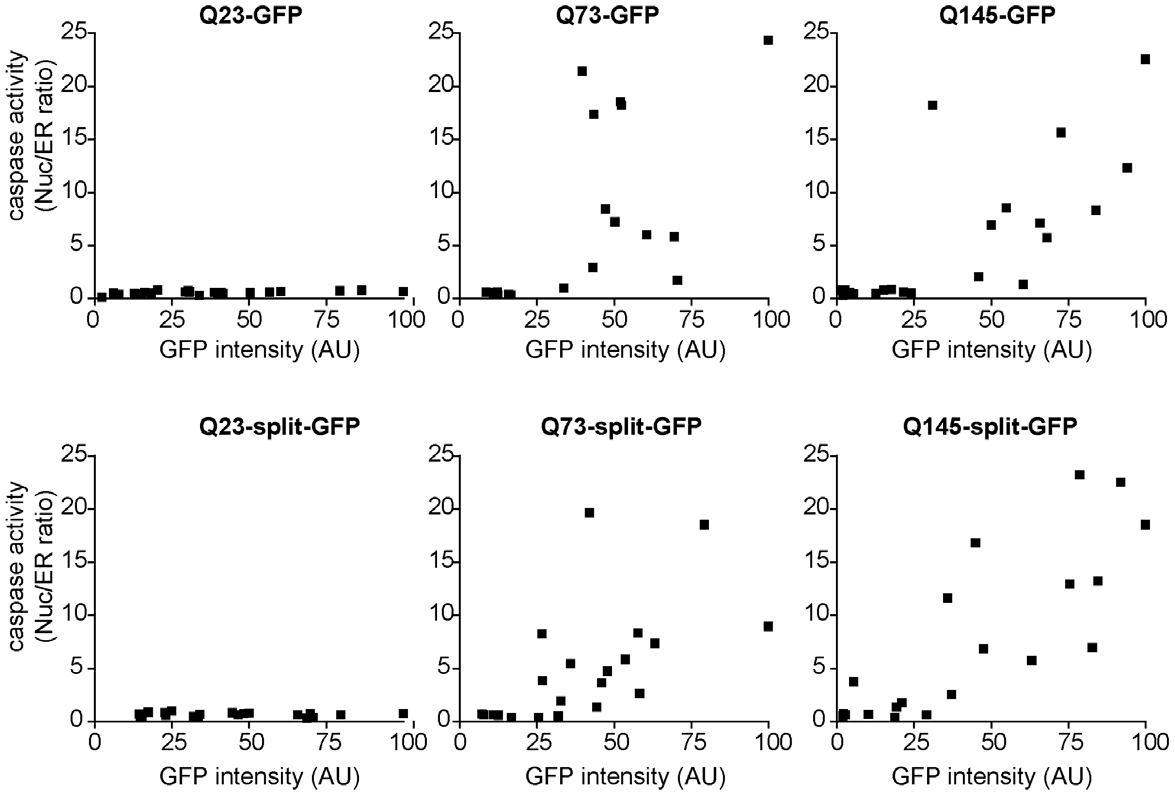
Levels of soluble cytoplasmic mHtt^ex1^ are a negative predictor of cell death. N2a cells were cotransfected with Q23, 73 or 145 Htt^ex1^ fused to GFP or split-GFP and ER-DEVD tdTomato for 48 h. GFP or split-GFP intensities and the nuclear/ER ratio of the apoptosis reporter were quantified for individual cells not presenting IBs. In each plot, the cell with the brightest GFP intensity was defined as having a mean intensity of 100 arbitrary units and other cell intensities were converted to this scale. Thus intensities in one plot are not directly comparable to intensities in another plot. Each square represents a single cell. Increasing mHtt^ex1^, but not wt Htt^ex1^, levels above some threshold were associated with increased cell death. The existence of apparent thresholds correlating with activation of the caspase activity suggests mHtt^ex1^ toxicity is sharply concentration dependent and may depend on titration of one or more key cellular factors.

With the split-GFP constructs, two trends immediately stand out. First, mHtt split-GFP expressing cells have substantially higher levels of caspase activity and this occurs in a much higher percentage of cells compared to cells expressing intact GFP constructs (Figs. 6C and D). Interestingly, the dramatically increased caspase activity was observed earlier than for cells expressing intact GFP constructs (Fig. 6C). Second, fusion of mHtt^ex1^ to split-GFP significantly increased the average caspase activity per cell regardless of the presence of IBs compared to cells expressing intact GFP fusions to mHtt^ex1^ (Figs. 6C and D). Soluble mutant polyglutamine expression levels of both the intact or split-GFP correlated with increased caspase activity (Fig. 7). Importantly, higher levels of soluble oligomers strongly correlate with increased cytotoxicity (Fig. 7). Caspase activity typically only occurred above some threshold level of any of the mHtt^ex1^ constructs (Fig. 7). That is, mHtt^ex1^ must achieve a critical concentration in cells to induce caspase activation. Taken together, our results strongly implicate soluble polyglutamine oligomers as an important (if not the predominant) toxic species in polyglutamine disease pathogenesis.

The intact and split-GFP construct results indicate IBs are neither required for toxicity nor do IBs appear to enhance polyglutamine toxicity. Notably, a high percentage of cells expressing mHtt^ex1^ split-GFP contained IBs and exhibited caspase activity (Fig. 6D). This contrasts with the report of Arrasate et al. [14], [18] describing how the presence of IBs formed from intact GFP mHtt^ex1^ constructs correlated with *enhanced* cell survival.

Could split-GFP, itself, cause cytotoxicity? We observe Q23 split-GFP also forms oligomers, but with no apparent cellular toxicity (Figs. 4C, D, and 6C). Split-GFP alone caused no apparent toxicity. As the only differences between Q23 and the Q73 and Q145 constructs are the numbers of polyglutamine repeats, we suggest cytotoxicity is due to oligomerization of extended polyglutamine proteins. Also, the use of split-GFP does not alter the trend of Htt^ex1^ toxicity, since mHtt^ex1^ fragments tagged to regular GFP are also toxic. However, increased mHtt^ex1^ oligomerization due to split-GFP does, in fact, result in an increase in the absolute toxicity of mHtt^ex1^ intermediate oligomers.

It is possible the split-GFP IBs could represent a novel cytotoxic species or could lack cytoprotective properties of intact GFP IBs. We consider it more likely that the rates of oligomer formation and IBs formation represent the relevant parameters for predicting cell survival outcomes. While we observed higher numbers of cells with split-GFP IBs and the rate of IBs formation was faster (Fig. 6B), mean caspase activity was not significantly higher than in cells with soluble cytoplasmic split-GFP extended polyglutamine constructs (Fig. 6C). This would suggest IBs could form at the time of or even after caspase activation. Furthermore, the constitutive production of split-GFP polyglutamine constructs with a high tendency to oligomerize could substantially increase the rate of soluble polyglutamine oligomer formation regardless of the presence of IBs, possibly overwhelming oligomer sequestration by the IBs formation pathway.

Formation of IBs and related aggresome structures is a cell regulated process. Relatively small oligomers or aggregates are specifically trafficked by the motor dynein along microtubules to the perinuclear microtubule organizing center [78]. There, cellular proteins, including p62, stimulate autophagic degradation of IBs [78]. We hypothesize that either sequestration of oligomers into IBs or the rate of autophagic clearance may not be sufficiently accelerated in the split-GFP system to cope with higher soluble oligomer levels. The split-GFP system now provides a powerful tool for investigating the kinetics and responsiveness of the IBs-autophagosome pathway to exceptionally high levels of soluble oligomers.

### Conclusions

Using two different and complementary approaches, we have followed changes in the oligomeric state of soluble mHtt^ex1^ in cells. The FRAP approach revealed increasing tendencies of pathogenic polyglutamine expansions to incorporate into progressively larger soluble complexes. These complexes are likely to include chaperones and potentially other proteins [75], [79], [80]. However, FRAP does not report whether complexes contain more than one mHtt^ex1^ molecule or even the general composition of complexes. Our FRAP results are consistent with the native gel results of Legleiter et al. [33] and Takahashi et al. [30], who observed greater than 50% of pathogenic polyQ constructs incorporate into complexes significantly larger than a nonpathogenic construct.

Split-GFP constructs revealed the capacity of all of the constructs to form oligomers of at least two mHtt^ex1^ molecules. However, the trapping ability of split-GFP prevents us from quantitating the fraction of dimer or higher oligomers that normally would exist at steady state. A recent study by Takahashi et al [30] reported detecting soluble complexes containing at least 3.5 monomers. Similarly, Ossato et al [81] measured oligomers of 5–15 mHtt^ex1^ molecules in cells, using a “number and brightness method.” Soluble oligomers never constituted more than 20% of the total soluble mHtt^ex1^ in the Ossato et al. study. However, our data suggest the *majority* (≥50%) of mHtt^ex1^-GFP reporters must be incorporated into complexes of significantly increased size to cause the observed decrease in *D* for FRAP and the slowed migration in native gels. This is because native gels and FRAP experiments are ensemble measurements, which detect relatively large changes for the entire population of proteins.

Previously, increasing levels of soluble polyglutamine oligomers in cells has required either expressing higher levels of polyglutamine proteins or regulating a co-factor of polyglutamine oligomerization. For example, Luo *et al.* stimulated oligomerization of mHtt fragments by overexpression of Pak1 [21]. Increased IBs formation and cell death followed, though the assay used did not directly investigate cell fates relative to the presence or absence of IBs. Split-GFP trapping of polyglutamine oligomers enabled us, for the first time, to directly demonstrate the higher propensity of soluble polyglutamine oligomers to form IBs and the increased toxicity of soluble oligomers in living cells without manipulating other cellular parameters. However, by artificially trapping monomer interactions and accelerating IBs formation, the split-GFP method increases the normal rate of conversion of monomers into oligomers and the flux of oligomer integration into IBs. Thus, the split-GFP method under-emphasizes any potential role monomer levels play in cell fate. While we cannot exclude the potential toxicity of mHtt^ex1^ monomers, our data establish that driving oligomerization clearly enhances the cytotoxic potential of mHtt^ex1^.

Taken together, an emerging picture of mHtt oligomerization in cells suggests all Htt^ex1^ constructs, including the nonpathogenic Q23, can form transient oligomers. Pathogenic polyQ constructs progressively incorporate into large complexes in cells, some of which include additional copies of polyQ constructs. Formation of complexes containing two or more copies of pathogenic polyQ molecules substantially impairs cell viability. Future studies will need to tease apart the relative toxicities of different soluble oligomers, i.e. do increasingly larger numbers of polyQ molecules in complexes increase cytotoxicities.

Our findings support the hypothesis that mHtt oligomers are indeed the toxic species in HD. Given the increasing body of evidence against a pathogenic role for IBs in polyglutamine diseases, we suggest therapeutics that block formation of soluble polyglutamine oligomers merit investigation. The split-GFP methodology is well suited for a noninvasive high throughput screen to identify compounds capable of inhibiting polyglutamine oligomerization in cells.

## Materials and Methods

### Cell lines

Neuro-2a (N2a), U-2 OS, and HEK 293t cells were obtained from ATCC. Cells were grown in 8-well Lab-tek chambers (Nunc; Rochester, NY) in RPMI media (Mediatech; Manassas, VA) containing 10% fetal bovine serum (Hyclone from Thermo Scientific; Rockford, IL), glutamine and penicillin/streptomycin (Invitrogen; Carlsbad, CA), in a 5% CO^2^ incubator at 37°C. N2a cells were routinely differentiated by incubating the cells with 5µM dbcAMP (N6′, 2′-*O*-dibutyrilaenosine-3′:5′-cyclic monophosphate sodium salt) (Sigma-Aldrich; St. Louis, MO) for two days.

### Constructs and transfection

Htt^ex1^Q23, Q73 and Q145 constructs were obtained from the Coriell Institute for Medical Research and the CHDI Foundation. Htt^ex1^ fragments were amplified by PCR using the following primers:

forward primer GATCAGATCTGCCACCATGGCGACCCTGGAAAAG

reverse primer GATCACCGGTCCTGGTCGGTGCAGCGG

and subcloned into the BglII and Age1 site of monomeric pEGFP-N1 vector.

Split-GFP constructs were generated by PCR using pEGFP-N1 as a template with the following primers:

Split-GFP 1–157 was amplified using the

forward primer GCAAATGGGCGGTAGGCG

reverse primer GATCGCGGCCGCTTACTGCTTGTCGGCCATG

and subcloned into Age1 and Not1 of SFpEGFP-N1. Split-GFP 158–238 was amplified using the

forward primer GATCACCGGTCCGGGAGCAAGAACGGCATCAAG

reverse primer GGTTCAGGGGGAGGTGT

and subcloned into Age1 and Not1 of pSuperfolderGFP-N1 [66].

The ER DEVD tdTomato vector was generated by PCR amplification of tdTomato [82] using the following primers:

forward primer GATCACCGGTATGGTGAGCAAGGGC

reverse primer GGTTCAGGGGGAGGTGT

and subcloned the resulting fragment into ER-DEVD-mStrawberry [77] using the Age1 and BsrG1 sites.

The Nalp1b allele 2 split-GFP was generated by PCR by amplifying Nalp1b using the

forward primer GATCGCTAGCGCCACCATGGAACAATCTCAG

reverse primer GATCCCCGGGCACCGGTACGCGTAGA

The resulting fragment was cloned into split-GFP-N1 vectors using the Nhe1 and Xma1 sites.

DNA constructs were transfected into N2a cells using Lipofectamine 2000 transfection reagent (Invitrogen) according to manufacturer instructions.

### Aggregation assay and quantification

N2a cells were plated and differentiated for two days prior transfection with Htt^ex1^-GFP or split-GFP constructs. Cells were imaged at various time points following transfection. Imaged were collected using fluorescence microscopy with a widefield microscope, Axiovert 200, (Carl Zeiss Microimaging Inc., Thornwood, NY) 63× oil NA 1.4 objective, 450–490 excitation/500–550 emission bandpass filter) and a Retiga 2000R camera (QImaging; Surrey, BC). The percentage of cells containing at least one or more IBs was quantified.

### FRAP and FLIP

Cells were imaged in phenol red–free RPMI supplemented with 10mM HEPES and 10% FBS. Live cells were imaged on a 37°C environmentally controlled chamber of a Duoscan confocal microscope system (Carl Zeiss Microimaging) with a 63× NA 1.4 oil objective and a 489 nm 100 mW diode laser with a 500–550 nm bandpass filter for GFP. FRAP and FLIP experiments were performed by photobleaching a region of interest at full laser power of the 489 nm line and monitoring fluorescence loss or recovery over time. No photobleaching of the adjacent cells during the processes was observed. Diffusion coefficient (*D*) measurements were calculated as described previously [36], [83].

### Photoactivation

N2a cells were transfected with Htt^ex1^-PA-GFP constructs for 24h and image on confocal microscope as previously described. Photoactivation experiments were performed by bleaching a small ROI within the cells with a 405 nm laser while constantly imaging cells with a 489 nm laser to detect activated state of PA-GFP.

### Quantitative fluorescence microscopy

Cells were fixed with freshly diluted 3.7% formaldehyde in PBS for 15 min at RT, permeabilized with PBS with 0.1% Triton X-100. Blocking was performed in 10% fetal bovine serum in 1× PBS. Subsequently, cells were labeled with anti-monomeric polyglutamine antibody 3B5H10 (Sigma Aldrich), followed by Alexa 555-conjugated anti-rabbit IgG secondary antibodies. Cells were imaged using fluorescence microscopy with an Axiovert 200 widefield microscope (Carl Zeiss Microimaging Inc.) with a 63× oil NA 1.4 objective, 450–490 excitation/500–550 emission bandpass filter. Image analysis was performed with ImageJ (National Institutes of Health; Bethesda, MD).

An important technical note is that the high fluorescence intensity of IBs saturates the CCD detector in the GFP channel, obscures the fluorescent signal of soluble GFP construct levels, and thus limits our ability to quantitate soluble GFP construct levels in IBs containing cells. Therefore, most of our study focuses on cells prior to the appearance of IBs. Alternatively, cells containing IBs are scored in general terms of having or not having IBs, without any attempt to quantitate absolute GFP intensity levels in those cells.

### Cell death

Cells were cotransfected with Htt^ex1^-GFP and ER-DEVD-tdTomato constructs. Alternatively, cells were transfected with ER-DEVD-tdTomato and treated for 3 h with 5 µM staurosporine (EMD Biosciences; Gibbstown, NJ). Fluorescent images were acquired with a widefield fluorescent microscope as previously described. For the ER DEVD-tdTomato, fluorescent intensity within the nucleus and the ER were obtained with ImageJ. Caspase activity was calculated by obtaining the ratio of mean fluorescence intensity of the nucleus divided by the intensity of the ER. Increase in caspase activity in mHtt expressing cells was quantified by calculating the % of cells with Nucleus/ER ratio higher than the average value obtained for Q23 Htt^ex1^-GFP.

### SDS-PAGE and immunoblot

N2a cells were plated in 12 well plates, differentiated and transfected with either Htt^ex1^ constructs fused to GFP or split-GFP. Cells were rinse twice with PBS and cells were lysed in 50 µl of sample buffer containing 1% SDS, 0.1 M Tris, pH8.0. Lysate were run on 12% Tris-tricine gels and transferred to nitrocellulose membrane, Antibodies used included anti-GFP and anti-RFP (generous gifts from Ramanujan S. Hegde), and HRP-labelled anti-rabbit (Jackson Immunoresearch Laboratories).

### Immunoprecipitations

For immunoprecipitations, N2a cells expressing Htt^ex1^ constructs in 6-well plates were washed twice with 1× PBS and lysed with IP buffer (1% Triton X-100, 50mM Hepes, pH 7.4, 100mM NaCl) containing EDTA-free protease inhibitor cocktail (Roche). Lysates were clarified for 10 min at maximum speed in a microcentrifuge at 4°C and incubated for 2 h at 4°C with agarose conjugated anti-GFP beads (MBL ltd.). The beads were washed four times in IP buffer, once in distilled water, eluted with SDS-PAGE sample buffer, and analyzed on 12% Tris-glycine gels, followed by blotting, staining, and development as for immunoblots.

### Native gel

N2a cells were plated in 12 well plates, differentiated and transfected with either Htt^ex1^ constructs fused to GFP or split-GFP. Cells were rinsed twice with native buffer (20mM Hepes, 150 mM NaCl and protease inhibitor cocktail). Then cells were lysed in cold native buffer containing 0.2% triton X-100. Sample were clarified by centrifugation and loaded on a 7.5% Tris-glycine native gel. Tris-glycine buffer was used as running buffer and gels were allowed to migrate for 4 h. Proteins were subsequently transferred on nitrocellulose membrane and processed for immunoblot for anti-GFP.

## Supporting Information

Figure S1 The mobility of Htt^ex1^ revealed by GFP photoactivation. N2a cells were transfected with Q23, 73 or 145 Htt^ex1^-PA-GFP for 24 h. Photoactivation a small ROI within the cytoplasm was performed with a 405 nm laser. By 5 s postactivation, the photoactivated pool of Htt^ex1^-PA-GFP has diffused throughout the entire cytoplasm. When IBs (bottom Q145 panels) were photoactivated, no significant redistribution of the mHtt^ex1^-PA-GFP to the rest of the cytoplasm was observed.
(TIF)

Figure S2 *D* values (µm^2^/s) of single cells transiently transfected with Htt^ex1^-GFP constructs containing 23, 73 or 145 polyQ repeats for 16 h and analyzed by FRAP in 293 and U-2 OS cells. * p<0.05 compared to Q23 Htt^ex1^-GFP.
(TIF)

Figure S3 (**A**) Comparison of Htt^ex1^-GFP and split-GFP fluorescence intensities. N2a cells were transfected for 24 h with Q73 mHtt^ex1^-GFP or split-GFP, fixed and immunofluorescently labeled with 3B5H10 antibody. Both constructs were imaged using the same exposure time for both channels. Subsequently exposure time was increased for split-GFP to reveal the GFP signal. Bar=20 µm. (**B**) Plot of *D* values and camera gain settings for N2a cells transiently expressing Q73 Htt^ex1^-GFP or Q73 Htt^ex1^-Split-GFP. Note that lower gain settings are used for brighter cells.
(TIF)

Figure S4 Validation of the ER-DEVD-tdTomato reporter functionality. N2a cells were transfected with ER-DEVD-tdTomato for 16 h and then treated with or without 5 µM staurosporine for 3h. The fluorescent intensity ratio of the nucleus over the ER calculated is presented in the plot. ** p<0.0001 compared to untreated cells. Bar=20 µm.
(TIF)

Figure S5 Comparison of Htt^ex1^ –GFP and Split-GFP constructs migration on native gel. Lysates from N2a cells cotransfected with Q23, 73 or 145 Htt^ex1^ fused to GFP or split-GFP were run onto a native gel and process for immunoblot with anti-GFP. Htt^ex1^ split-GFP constructs forms higher molecular weigh complexes than those fused to intact GFP. Interestingly, when Q23s157 is coexpressed with Q23-GFP, interaction is not possible and this is reflected by its inability to form higher complexes observed when expressed with the Q23 s238.
(TIF)
