# Supplementary figures and images for: Formation and Toxicity of Soluble Polyglutamine Oligomers in Living Cells
Source: PLoS One. 2010 Dec 28;5(12):e15245. doi: 10.1371/journal.pone.0015245 (PMC3011017; doi:10.1371/journal.pone.0015245)

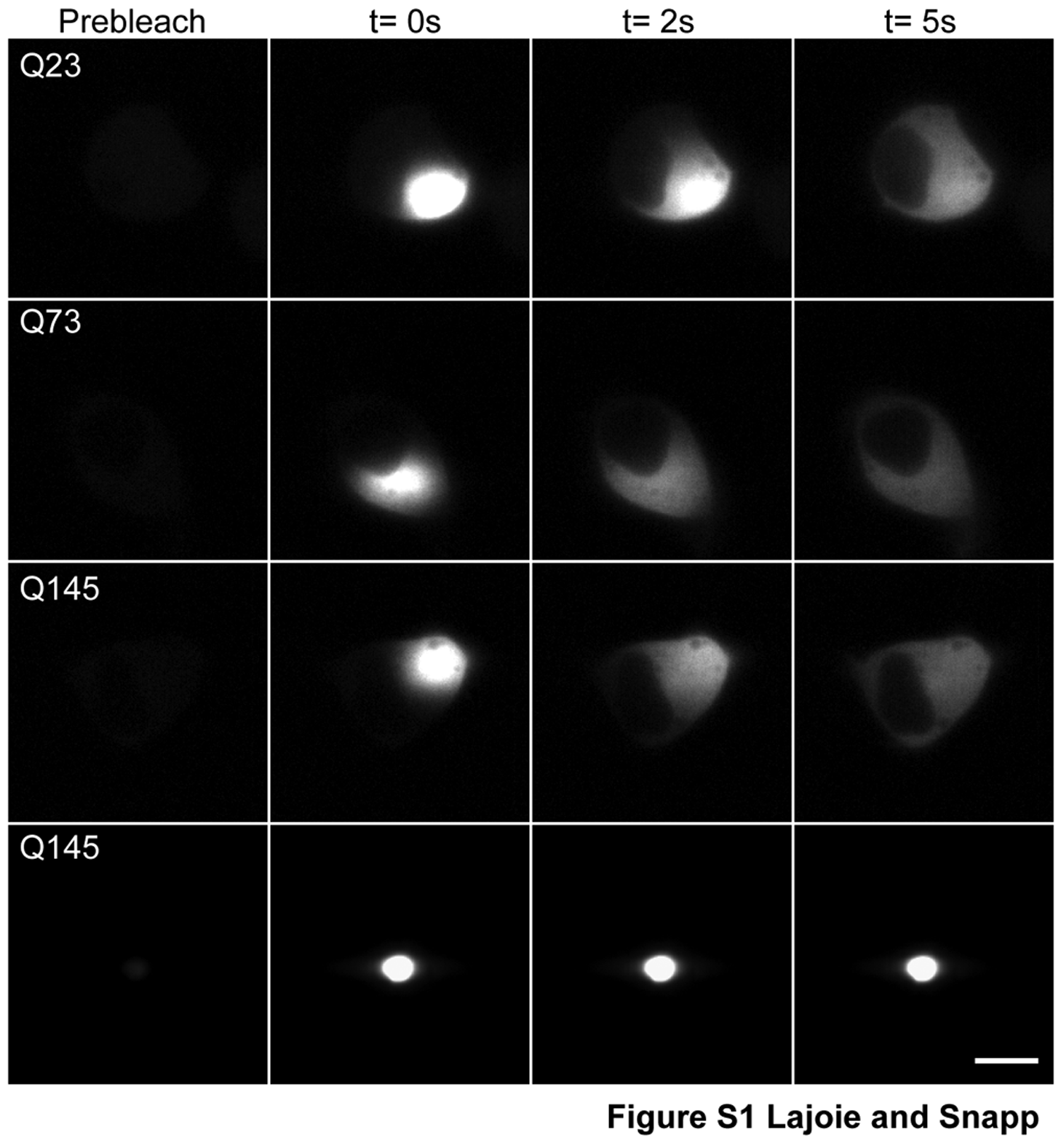

Supplement: Figure S1 — The mobility of Httex1 revealed by GFP photoactivation. N2a cells were transfected with Q23, 73 or 145 Httex1-PA-GFP for 24 h. Photoactivation a small ROI within the cytoplasm was performed with a 405 nm laser. By 5 s postactivation, the photoactivated pool of Httex1-PA-GFP has diffused throughout the entire cytoplasm. When IBs (bottom Q145 panels) were photoactivated, no significant redistribution of the mHttex1-PA-GFP to the rest of the cytoplasm was observed. (TIF) [file pone.0015245.s001.tif]

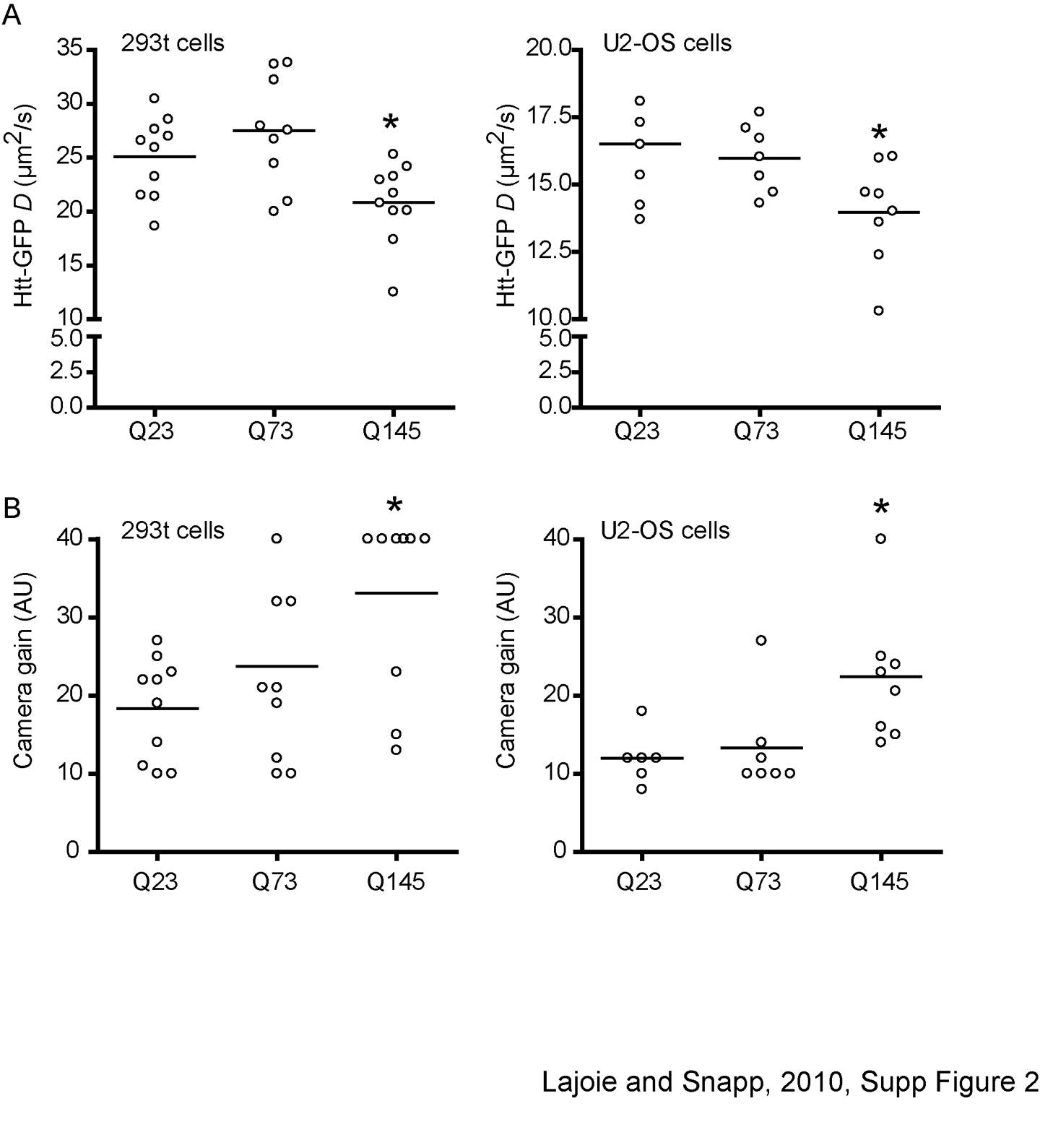

Supplement: Figure S2 — D values (µm2/s) of single cells transiently transfected with Httex1-GFP constructs containing 23, 73 or 145 polyQ repeats for 16 h and analyzed by FRAP in 293 and U-2 OS cells. * p<0.05 compared to Q23 Httex1-GFP. (TIF) [file pone.0015245.s002.tif]

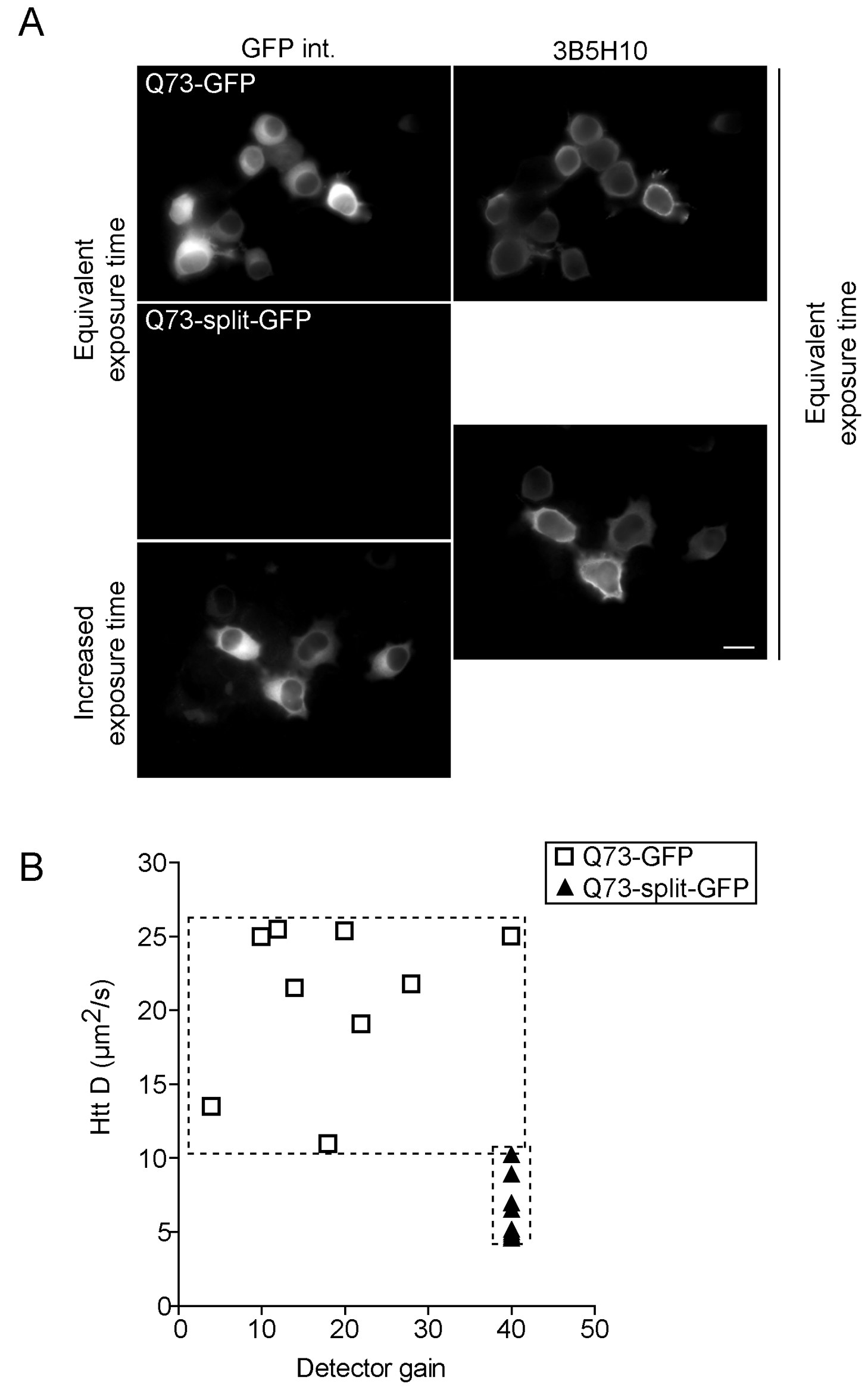

Supplement: Figure S3 — (A) Comparison of Httex1-GFP and split-GFP fluorescence intensities. N2a cells were transfected for 24 h with Q73 mHttex1-GFP or split-GFP, fixed and immunofluorescently labeled with 3B5H10 antibody. Both constructs were imaged using the same exposure time for both channels. Subsequently exposure time was increased for split-GFP to reveal the GFP signal. Bar=20 µm. (B) Plot of D values and camera gain settings for N2a cells transiently expressing Q73 Httex1-GFP or Q73 Httex1-Split-GFP. Note that lower gain settings are used for brighter cells. (TIF) [file pone.0015245.s003.tif]

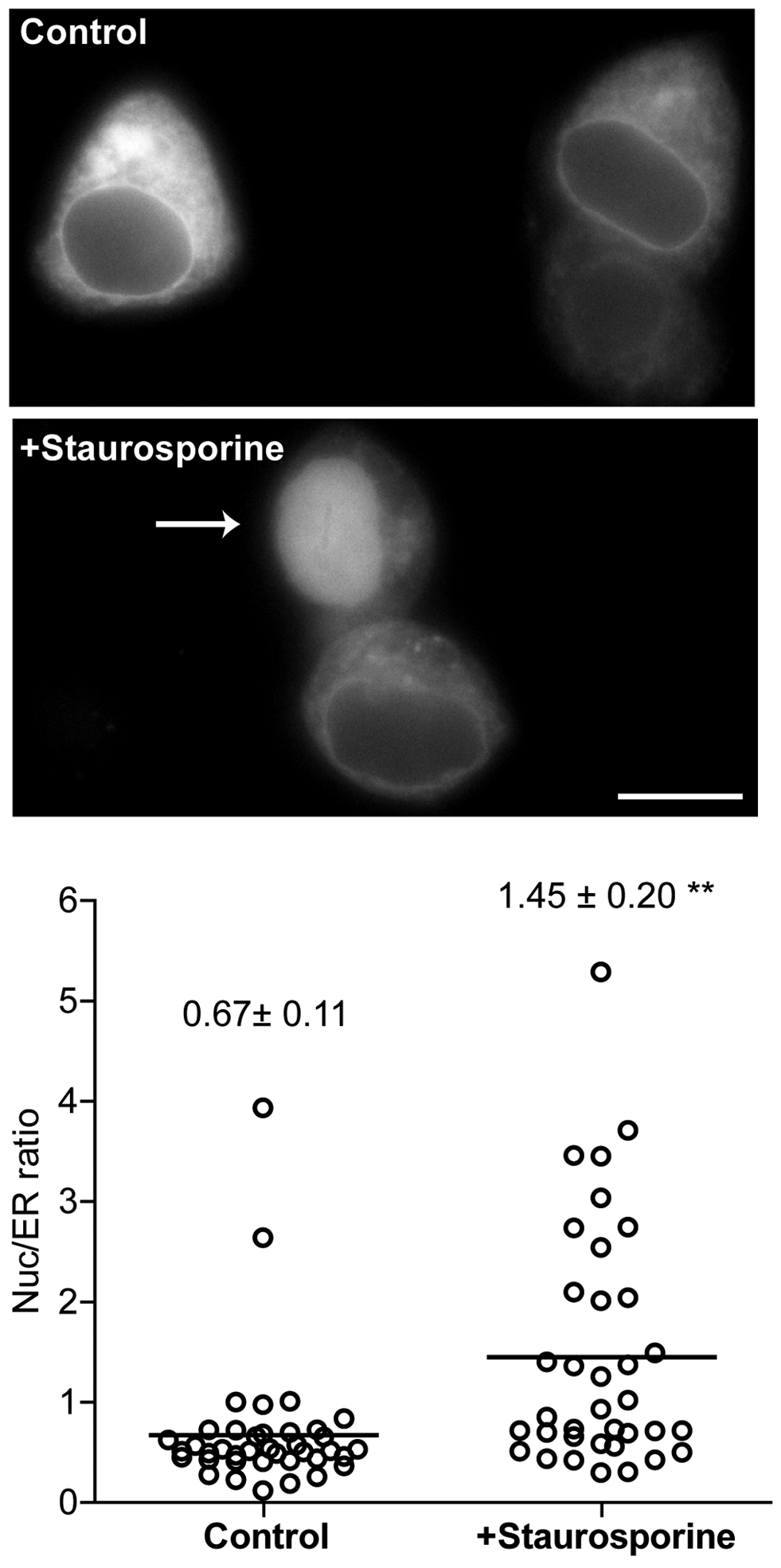

Supplement: Figure S4 — Validation of the ER-DEVD-tdTomato reporter functionality. N2a cells were transfected with ER-DEVD-tdTomato for 16 h and then treated with or without 5 µM staurosporine for 3h. The fluorescent intensity ratio of the nucleus over the ER calculated is presented in the plot. ** p<0.0001 compared to untreated cells. Bar=20 µm. (TIF) [file pone.0015245.s004.tif]

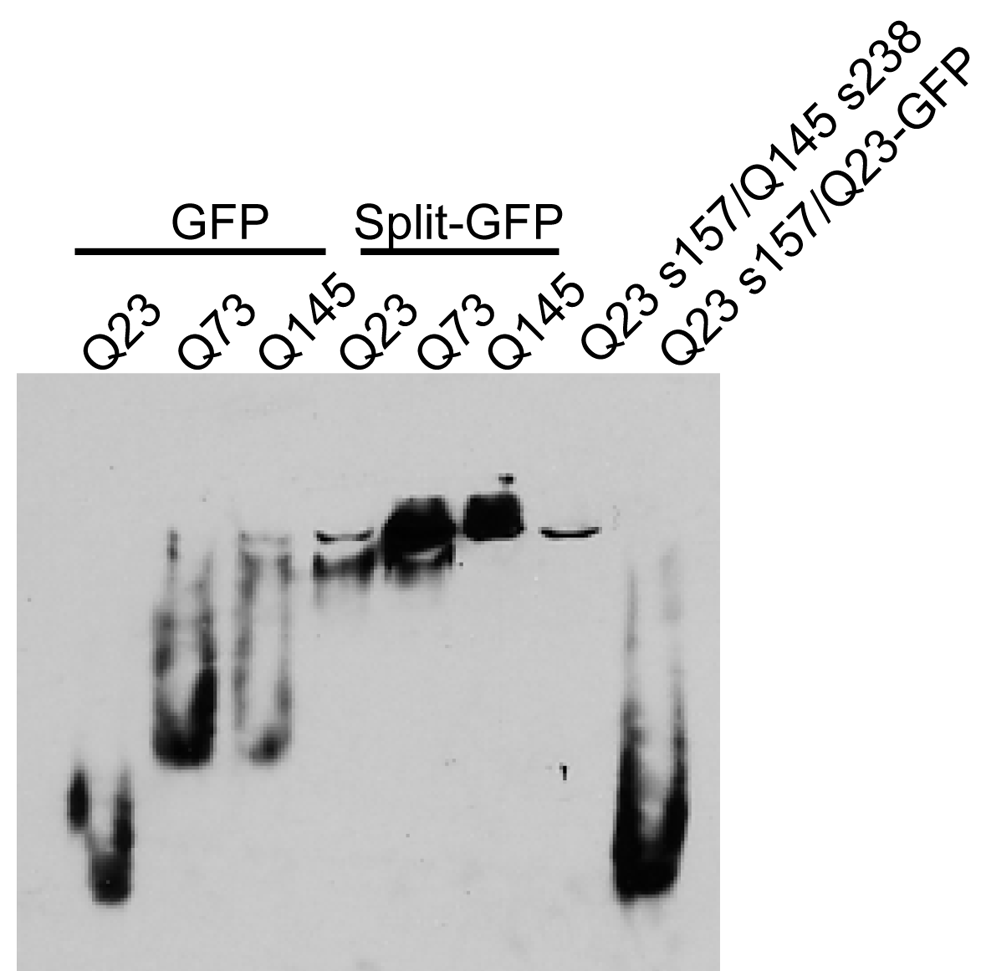

Supplement: Figure S5 — Comparison of Httex1 –GFP and Split-GFP constructs migration on native gel. Lysates from N2a cells cotransfected with Q23, 73 or 145 Httex1 fused to GFP or split-GFP were run onto a native gel and process for immunoblot with anti-GFP. Httex1 split-GFP constructs forms higher molecular weigh complexes than those fused to intact GFP. Interestingly, when Q23s157 is coexpressed with Q23-GFP, interaction is not possible and this is reflected by its inability to form higher complexes observed when expressed with the Q23 s238. (TIF) [file pone.0015245.s005.tif]
